# Are CB2 Receptors a New Target for Schizophrenia Treatment?

**DOI:** 10.3389/fpsyt.2020.587154

**Published:** 2020-10-30

**Authors:** Isadora L. Cortez, Naielly Rodrigues da Silva, Francisco S. Guimarães, Felipe V. Gomes

**Affiliations:** Department of Pharmacology, Ribeirão Preto Medical School, University of São Paulo, Ribeirão Preto, Brazil

**Keywords:** cannabinoids, endocannabinoid system, psychosis, dopamine, microglia

## Abstract

Schizophrenia is a complex disorder that involves several neurotransmitters such as dopamine, glutamate, and GABA. More recently, the endocannabinoid system has also been associated with this disorder. Although initially described as present mostly in the periphery, cannabinoid type-2 (CB2) receptors are now proposed to play a role in several brain processes related to schizophrenia, such as modulation of dopaminergic neurotransmission, microglial activation, and neuroplastic changes induced by stress. Here, we reviewed studies describing the involvement of the CB2 receptor in these processes and their association with the pathophysiology of schizophrenia. Taken together, these pieces of evidence indicate that CB2 receptor may emerge as a new target for the development of antipsychotic drugs.

## Introduction

Schizophrenia is a highly disabling psychiatric disorder of multifactorial etiology that affects about 1% of the world population (1). The symptoms of schizophrenia are divided into three main groups: positive, negative, and cognitive symptoms. Positive symptoms are characterized by an exaggeration of normal functions, presenting mainly as hallucinations, delusional ideas, defragmentation of thought, and psychomotor agitation. On the other hand, the negative symptoms are characterized by a loss of normal functions, leading to affective blunting, anhedonia, and social withdrawal (2). The cognitive symptoms are related to deficits in domains such as working memory, attention, verbal learning and memory, problem-solving, among others (3).

Although the pathophysiology of schizophrenia remains mostly unknown, it has long been thought that it involves an imbalance among several neurotransmitter systems. The first, and likely the most influential, hypothesis about the neurobiology of schizophrenia proposes that changes in the dopamine system, mainly a striatal hyperdopaminergic state, would be responsible for the psychotic symptoms (4). Following this initial proposal, it was later suggested that negative and cognitive symptoms would be associated with a hypodopaminergic state in the prefrontal cortex (PFC) (5).

The first drugs used to treat schizophrenia, known as typical antipsychotics, act as antagonists at dopamine D2 receptors. Besides their effects on positive symptoms, they also cause adverse effects such as extrapyramidal side effects and hyperprolactinemia, resulting in a high discontinuation rate. The second-generation or atypical antipsychotics, despite also targeting dopamine D2 receptors, also bind to receptors associated with other neurotransmitter systems (6). Although these drugs have a lower tendency to induce adverse motor effects at therapeutic doses than first-generation antipsychotics, they are associated with undesirable effects that may limit their use, such as metabolic changes and weight gain (7, 8). In addition, while positive symptoms have a good clinical response to typical and atypical antipsychotics, the negative and cognitive impairments are more resistant to the available drugs. Together, these observations support the urgent need to develop new drugs with better efficacy and tolerability (9–11).

Considering the lack of therapeutic options and the complexity of this disorder, recent hypotheses have emerged involving other neurotransmitter systems such as the glutamatergic, serotonergic, gamma-aminobutyric acid (GABA), and, more recently, the endocannabinoid (12–16).

## The Endocannabinoid System

The endocannabinoid system (ECS) is a modulatory system that plays a crucial role in brain development, synaptic plasticity, and response to endogenous and environmental insults (17). The ECS comprises endogenous cannabinoids (endocannabinoids), cannabinoid receptors, and the enzymes responsible for the synthesis and degradation of endocannabinoids. The two main and best-characterized endocannabinoids are N-arachidonoyl ethanolamine (anandamide) and 2-arachidonoyl glycerol (2-AG) which, unlike most classical neurotransmitters, are produced on demand. There are reports, however, indicating that they might also be stored intracellularly (18, 19).

In the central nervous system (CNS) anandamide and 2-AG are synthesized and secreted from postsynaptic neurons. They bind to cannabinoid CB1 and CB2 receptors located on presynaptic terminals, acting as retrograde messengers and to CB2 receptors located on the postsynaptic site of some neurons (20). Once released in the synaptic cleft, endocannabinoids can be taken up by specific transport proteins and then broken down by the fatty acid amid hydrolase (FAAH) and monoacylglycerol lipase (MAGL) enzymes, which degrades anandamide and 2-AG, respectively (21, 22).

Although the effects of endocannabinoids are mediated mainly by CB1 and CB2 cannabinoid receptors, others receptors such as the peroxisome proliferator-activated receptors (PPARs) and transient receptor potential (TRP) channels, can also be activated by these compounds (17, 23). CB1 and CB2 receptors are G-protein-coupled receptors (GPCRs) that, in addition to interacting with endocannabinoids, are also activated by synthetic and plant-derived cannabinoids.

The cannabinoid receptors are G protein-coupled receptors (GPCR), which couple mainly to the G_i_ and G_0_ classes of G proteins. Their activation inhibits the adenylyl cyclases enzymes, activates mitogen-activated protein kinases and modulates voltage-dependent ion channels (i.e., activating voltage-dependent potassium channels and inhibiting voltage-dependent calcium channels) (23). Overall, the intracellular signaling induced by the activation of cannabinoid receptors inhibits neurotransmitter release (17).

CB1 receptors are the most prevalent GPCR in the CNS and are located mainly in the cortex, hippocampus, amygdala, basal ganglia, and cerebellum (24). This receptor is the major mediator of the psychoactive effects of the *Cannabis sativa* plant and its derivatives. Many studies investigating cannabis abuse and psychosis have prompted debates as to whether the ECS is involved in the pathophysiology of schizophrenia (25). By acting on cannabinoid CB1 receptors, THC, the main cannabinoid found in cannabis and responsible for the majority of its psychotropic effects, interferes with brain maturation and causes long-lasting neurobiological changes when chronically administered (26, 27). THC also influences the release of neurotransmitters, such as dopamine and glutamate, that are involved in the pathophysiology of schizophrenia (28). Moreover, during adolescence, cannabis abuse has been associated with an increased risk for schizophrenia development (29). Corroborating this observation, other results also support the involvement of CB1 receptors in schizophrenia. For example, genetic associations between polymorphisms of CB1 receptors and other ECS-related genes have been related to a higher susceptibility to schizophrenia (30, 31) and response to antipsychotic drugs (32–34). Moreover, increased binding of CB1 receptor ligands has been found in the post-mortem brain of schizophrenia patients (35). It is noteworthy, however, that negative and controversial findings have also been found. For example, whereas increased levels of anandamide in the cerebrospinal fluid have been described in the prodromal stage of psychosis and antipsychotic-naïve first-episode psychosis patients (36, 37), a decrease in endocannabinoid synthesizing enzymes (NAPE and DAGL) was found in first-episode (38). These controversial data suggest that the ECS involvement in schizophrenia is complex and far from being completely understood (36, 39–41). Also, there is a lack of studies investigating changes in the ECS at different stages of the disorder.

## The CB2 Receptor

The CB2 receptor shares 44% homology with the CB1 receptor (23, 42). Early studies suggested that CB2 receptors were not present in the brain but highly expressed in peripheral tissues, particularly in the immune system. Therefore, these receptors became a target for developing new pharmacological therapies to inflammatory pathological conditions, including pain, autoimmune, and neurodegenerative disorders (43–46). With the development of increasingly selective and sensitive tools, it was possible to identify CB2 receptors throughout the CNS.

CB2 receptors are expressed in the brain at lower levels than CB1 receptors, being present in glial cells, such as microglia and astrocytes, and specific subpopulations of neurons (20, 47–51). In neurons, unlike CB1, CB2 receptors are mainly expressed at postsynaptic levels, which could contribute to some of the opposite effects found after their activation (20). For example, while presynaptic CB1 receptor activation in GABAergic neurons increases the probability of postsynaptic neuronal excitation, by decreasing GABA, the activation of postsynaptic CB2 receptors usually inhibits neuronal excitability (52, 53). However, CB2 receptors located in presynaptic terminals have also been described, where, similar to CB1 receptors, they modulate neurotransmitter release (54).

Another unique feature of CB2, compared to CB1 receptors, is that they are inducible and upregulated in glial cells in response to various insults, including inflammation and chronic pain (55). In glial cells, the activation of CB2 receptors inhibits the release of several inflammatory mediators, including nitric oxide and pro-inflammatory cytokines such as interleukin (IL)-1, tumor necrosis factor (TNF)-α, and IL-6, and increases the release of anti-inflammatory cytokines such as IL-10 and IL-1 receptor antagonist (56, 57). Also, CB2 receptors modulate the activation, proliferation, differentiation, and migration of microglia (58–60). Due to the presence of CB2 receptors in both glial cells and neurons, several groups have investigated the role of these receptors in neuroinflammation and neuroprotection (44, 56, 61, 62), and as potential targets to treat chronic neurodegenerative disorders, such as Alzheimer's, Parkinson's, and Huntington's disease (61, 63), and psychiatric disorders, such as schizophrenia and depression (52, 64–68). A wealth of evidence indicates that inflammatory/immune changes are associated with these disorders (69, 70).

## CB2 Receptors and Schizophrenia

Accumulating evidence points that CB2 receptor-related changes are present in schizophrenia. An increase in the frequency of two single nucleotide polymorphisms (SNP) in the CB2 receptor gene (rs12744386 and rs2501432), which decrease the function of these receptors, was described in schizophrenia patients (71). More recently, a genome-wide association study of more than 120,000 participants identified an SNP intronic to the CB2 receptor gene highly associated with distressing psychotic experiences (72). In addition, non-treated first-episode psychosis and acute schizophrenia patients treated with antipsychotics showed a decreased peripheral expression of CB2 receptors than to healthy controls (38, 40). However, there has been a lack of post-mortem and neuroimaging studies evaluating the expression of CB2 receptors in patients with schizophrenia.

The preclinical studies suggesting the involvement of CB2 receptors in key neurotransmitter systems associated with schizophrenia have been recently reviewed (64). In the present paper, in addition to address these studies, we further discuss the role of CB2 receptors in inflammatory and stress-associated neuroplastic processes that have also been associated with this disorder.

### CB2 Receptors in Animal Models of Schizophrenia Based on Dopamine Dysregulation

Dysregulation of the midbrain dopamine system, characterized mainly by a striatal hyperdopaminergic state, is a hallmark of the pathophysiology of schizophrenia (73). This hyperdopaminergic state is implicated in psychotic symptoms, which involve perceptual disturbances (hallucinations) and fixed beliefs resistant to contradictory evidence (delusions).

Excitatory, inhibitory, and modulatory inputs control the dopamine neurotransmission by modifying its release, postsynaptic effects, and neuronal firing patterns (74). In general, whereas glutamatergic inputs onto dopamine neurons increase excitability, GABAergic inputs inhibit dopamine neuronal function (75, 76). In addition, autoregulation of dopamine release can occur through presynaptic D2 receptors. The activation of these receptors results in inhibitory feedback that decreases dopamine release (77).

Several studies indicate that the ECS modulates the midbrain dopamine system and dopamine-related behaviors (78–80). These studies have mainly focused on CB1 receptors because, as discussed above, CB2 receptors have long been considered as peripheral cannabinoid receptors (42). CB1 receptors are expressed at low to moderate levels throughout the mesolimbic dopamine pathway. They are also highly expressed in the medial PFC (24), where they can modulate dopamine transmission (81). In the ventral tegmental area (VTA), CB1 receptors are expressed presynaptically in glutamatergic and GABAergic terminals, modulating dopamine efflux in striatal regions (82). Based on this evidence, the CB1 receptor was proposed as a promising target for treating psychiatric disorders associated with dopamine dysregulation, such as schizophrenia and drug abuse (83). However, studies with the CB1 receptor antagonist rimonabant, although yielding to promising findings on psychostimulant addiction (84), revealed that this drug induces significant adverse effects, including depression and suicide ideation (85), which limited its therapeutic use.

Similar to CB1, CB2 receptors also modulate the dopamine system. Animals lacking CB2 receptors (CB2KO) present a decrease in basal motor activity, disruption in the prepulse inhibition (PPI) test, cognitive impairments, and enhanced response to acute cocaine (66). This behavioral profile is commonly associated with symptoms of schizophrenia. Chronic treatment with the second-generation antipsychotic risperidone attenuated the PPI deficits in CB2KO mice (66). Besides, the pharmacological blockade of CB2 receptors in the nucleus accumbens (NAc) by the local infusion of the CB2 receptor antagonist AM630 increased locomotor activity and extracellular NAc dopamine levels in wild-type and CB1 receptor knockout (CB1KO), but not in CB2KO mice (79). On the other hand, similar to antipsychotics (86), drugs that activate CB2 receptors, such as the CB2 receptor agonist JWH133, attenuate cocaine-induced increased locomotor activity and its rewarding properties (87). Also, Xi et al. (79) found that JWH133, in a dose-dependent manner, inhibited cocaine self-administration, and cocaine-enhanced locomotion and NAc dopamine levels in wild-type and CB1KO, but not in CB2KO mice. In addition, JWH133 prevented the acquisition and expression of cocaine sensitization in mice. Both effects were blocked by the CB2 receptor antagonist AM630 (88). Overall, these pieces of evidence indicate that CB2 receptors modulate dopamine function and its related behaviors. However, the mechanisms by which this modulation occurs are not yet completely clear.

CB2 receptors are present on the cell body of dopamine neurons in the VTA and on the terminal of these neurons in the NAc (89–91), where they can colocalize with D2 receptors (89). Functionally, mice with a selective deletion of CB2 receptors in VTA dopamine neurons (DAT-Cnr2 cKO) present a greater locomotor response to the acute administration of amphetamine and cocaine than wild-type animals (78). DAT-Cnr2 cKO mice also show enhanced cocaine-induced conditioned place preference and stereotypical behaviors, indicating that these receptors play a role in the VTA (92). Also, behavioral changes associated with the negative symptoms of schizophrenia were found in DAT-Cnr2 cKO mice, including anhedonia and enhanced behavioral despair (92). On the other hand, mice overexpressing CB2 receptors display an opposite behavioral profile, with lower locomotor response, self-administration, and place preference caused by cocaine (89).

**Figure 1:**
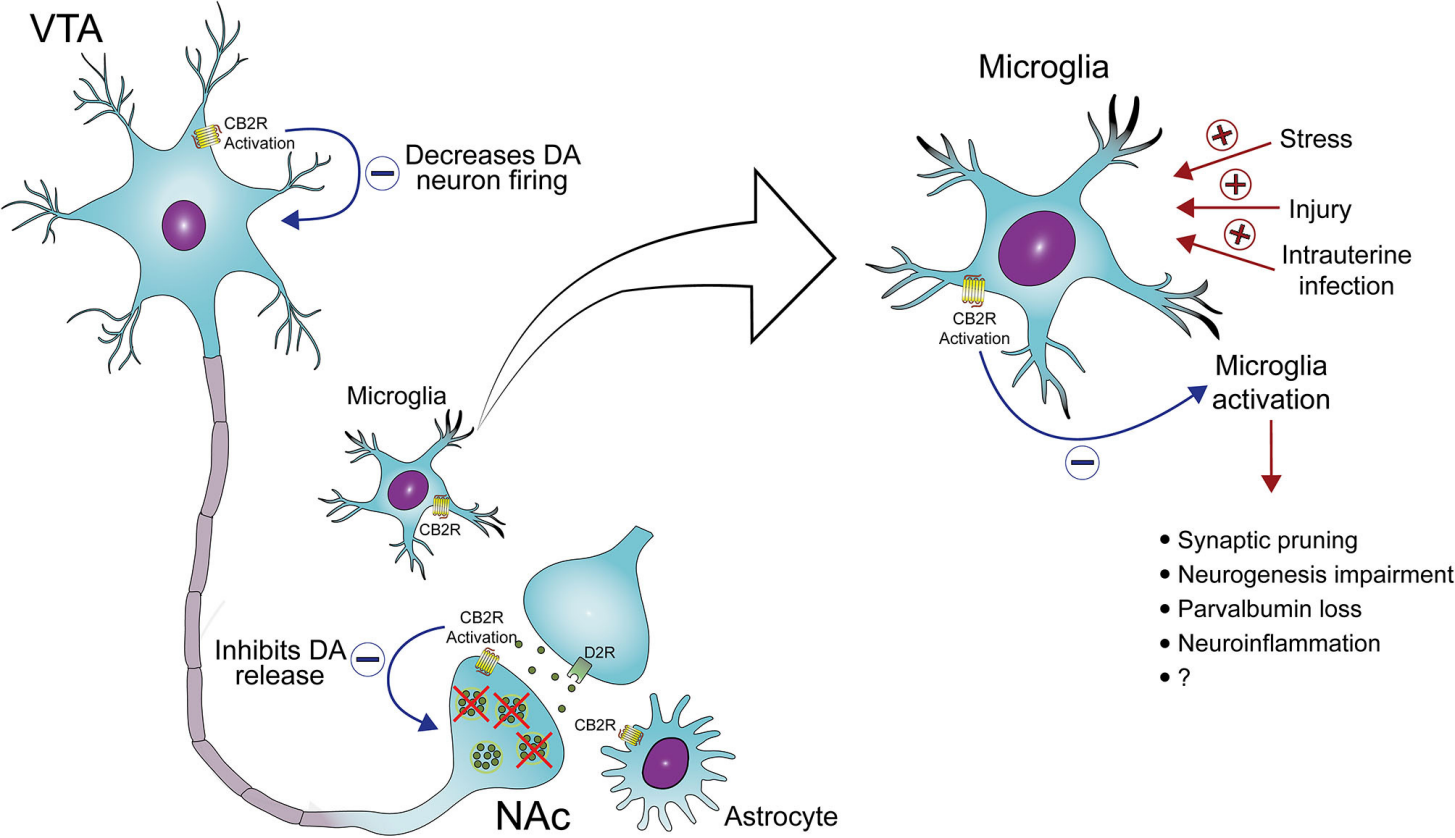
CB2 receptors as a target to treat midbrain dopamine system dysregulation and neuroinflammation in schizophrenia. The activation of CB2 receptors located in the cell body of ventral tegmental area (VTA) dopamine (DA) neurons and the terminal of these neurons in the nucleus accumbens (NAc) decreases DA neuron firing and DA release, respectively. In addition, the activation of CB2 receptors in microglia decreases the release of pro-inflammatory mediators and, possibly, microglia-mediated neurotoxicity. Several risk factors for schizophrenia, such as stress and maternal immune activation, lead to microglia activation, which has been associated with abnormal synaptic pruning, neurogenesis impairment, deficits in parvalbumin expression, and neuroinflammation, all common findings in schizophrenia.

In the VTA, CB2 receptors expressed in dopamine neurons can modulate dopamine neuronal excitability. Electrophysiological studies indicated that activation of CB2 receptors by JWH133 inhibits VTA dopamine neurons firing *in vivo* and *ex vivo*. Also, the infusion of this CB2 receptor agonist into the VTA and NAc inhibited cocaine self-administration and cocaine-enhanced extracellular dopamine levels. These effects were not seen in CB2KO mice and after the pretreatment with a CB2 receptor antagonist in wild-type mice (90, 93). JWH133 also decreased glutamatergic synaptic transmission in VTA dopamine neurons. However, the pharmacological blockade of synaptic transmission did not prevent the inhibitory effect of JWH133 on dopamine neuronal activity (93). Therefore, CB2 receptor activation does not impair the glutamatergic excitatory input to dopamine neurons and could directly modulate VTA excitability. Corroborating this possibility, the activation of postsynaptic CB2 receptors (a G_i/o_-coupled receptor) in VTA dopamine neurons reduces intracellular cAMP levels and enhances K^+^ channel function, decreasing the excitability of these neurons (93). In addition, Foster et al. have recently shown that the activation of muscarinic M4 receptors on D1 receptor-spiny projection neurons increases the release of 2-AG. Through the activation of CB2 receptors located in presynaptic terminals of dopamine neurons, this endocannabinoid causes a sustained inhibition of dopamine release. The authors have also described that the activation of M4 receptors reverses PPI disruption, an effect blocked by CB2 receptor antagonism (94). Taken together, these results indicate that CB2 receptors modulate dopaminergic transmission and, therefore, could be a promising target for the treatment of mental disorders associated with dopamine dysregulation, such as drug abuse and schizophrenia (Figure 1) (64, 66, 68, 80). Additional studies are needed to fully elucidate the modulatory role of CB2 receptors on dopamine function and how their pharmacological manipulation could help treat psychiatric disorders such as schizophrenia. Moreover, the impact of repeated treatment with CB2 receptor agonists on dopaminergic neurotransmission also needs to be further investigated.

### CB2 Receptors in Animal Models of Schizophrenia Based on NMDA Receptor Hypofunction

Ketamine and phencyclidine (PCP) induce schizophrenia-like signs in healthy subjects (95) and exacerbate schizophrenia symptoms in schizophrenia patients (96). Since ketamine and PCP act mainly as NMDA receptor antagonists, these clinical observations led to the proposal that a hypofunction of NMDA receptors may underlie schizophrenia symptoms. Unlike drugs that enhance dopamine neurotransmission, which induce only psychotic symptoms, ketamine and PCP evoke behavioral changes associated with not only the positive but also the negative and cognitive symptoms observed in schizophrenia patients (96). In rodents, acute or repeated administration of NMDA receptor antagonists such as ketamine, PCP, and MK-801, have been used to model schizophrenia (97). The schizophrenia-like signs induced by these drugs are proposed to depend on NMDA receptors blockade in parvalbumin containing inhibitory GABAergic interneurons (98, 99). A decrease in parvalbumin expression is one of the most robust findings in post-mortem brains of schizophrenia patients (100). This decrease is also described in several animal models of schizophrenia (101), including those based on NMDA receptor hypofunction (102, 103). The functional loss of theses interneurons could result in the dopamine dysregulation and cognitive deficits seen in schizophrenia.

The acute administration of NMDA receptor antagonists induces hyperlocomotion and PPI deficits in rodents. CB2 receptor agonists were found to either attenuate or reverse these changes. For example, the CB2 receptor agonist JWH105 reversed MK-801-induced PPI deficits. Supporting the involvement of CB2 receptor, JWH105 effects were blocked by the CB2 receptor antagonist AM630, but not by the CB1 receptor antagonist AM251 (104). As expected, contrary to the effects of the CB2 receptor agonists, the blockade of CB2 receptors exacerbates both the PPI impairments and increased the locomotor activity induced by MK-801 (71, 105).

Numerous preclinical and clinical studies have indicated that cannabidiol (CBD), the major nonpsychotomimetic compound found in the Cannabis plant, presents antipsychotic properties (106). Several pharmacological targets have been suggested to mediate CBD effects (107), including CB2 receptors (108, 109). In a recent work from our group, however, a CB2 receptor antagonist failed to reverse the positive effects of CBD on the memory and social interaction deficits caused in mice by repeated treatment with MK-801 (110). In this study CBD was administered after the treatment with NMDA receptor antagonist. In a previous study we found that CBD prevents the behavioral deficits and microglial activation caused by 28 days of daily MK-801 administration (111). The involvement of CB2 receptors in this preventive effect has not yet been investigated.

### CB2 Receptors as Targets for Controlling a Pro-inflammatory State in the Schizophrenic Brain

Besides the widely accepted hypotheses based on dysfunctions in dopamine and GABA-glutamate systems, dysregulation of the immune system has also been associated with the pathophysiology of schizophrenia (112).

In a healthy brain, constitutive cytokines play an important role in physiological and functional processes such as brain development, neurotransmission, and cognition (113–115). Under normal and pathological conditions in the CNS, cytokines are produced mainly by microglia and astrocytes (116, 117). Microglia are the CNS resident macrophages and play an important role in innate immunity, rapidly responding to any pathological changes in the brain. In normal conditions, microglia contributes to synaptic development and plasticity promotes neuronal survival, and always monitors the environment by continually moving their processes (118). Prolonged microglia activation might cause brain injuries. For example, increased microglia activation during brain development may lead to abnormal synaptic pruning, which has been associated with schizophrenia (119). Besides, increased microglia activation may result in expression deficits in parvalbumin containing interneurons and in their perineuronal nets (120).

Schizophrenia patients show increased serum levels of pro-inflammatory cytokines such as IL-2, IL-6, and IL-8 (121, 122). Elevated IL-1β levels were also found in the cerebrospinal fluid of drug-naïve patients (123). Moreover, infections during the perinatal period lead to maternal immune activation characterized by a marked increase in pro-inflammatory cytokines. It may disrupt neurodevelopmental processes in the fetus and be associated with a greater risk for schizophrenia development (124–126).

Increased microglia density and in markers of microglia activation have been reported in the post-mortem brain of schizophrenia patients (127). In addition, neuroimaging studies have revealed an overactivated state of microglia in schizophrenia patients (128, 129). This state has been correlated with positive symptoms and disease duration (130). Thus, the appropriate control of microglial activation might be a promising therapeutic strategy for schizophrenia. In accordance with this proposal, some reports have demonstrated antipsychotic-like effects of minocycline, an inhibitor of microglial activation. Adjunctive therapy of minocycline to antipsychotics was beneficial in animal models and schizophrenia patients, especially against negative symptoms (131–134). Other studies, however, have failed to show any beneficial effect of this treatment (135). Furthermore celecoxib, an anti-inflammatory drug, used as an add-on medication to antipsychotics chronic schizophrenia effectively treated positive symptoms (136, 137). Taken together, these studies suggest that, even if it is still unknown whether the immune dysfunction seen in schizophrenia is a primary factor or a secondary consequence, controlling this dysfunction could be beneficial.

The expression of CB2 receptors in microglia is modified depending on their activation, being low in the healthy brain, and high under pathological conditions (138, 139). Several studies indicate that CB2 receptor activation inhibits microglia-mediated neurotoxicity and reduces pro-inflammatory cytokine levels (140). When exposed to injury or infection, the resident microglia, similar to what occurs with macrophages, polarizes toward a pro-inflammatory phenotype (M1), characterized by the production of pro-inflammatory cytokines and antigen presentation. After activation, the M2 phenotype facilitates the resolution of the inflammatory state, through anti-inflammatory cytokines, establishing homeostasis (141). CB2 receptor activation facilitates microglia transformation from M1 to M2 phenotype, leading to a reparative scenario (142). On the other hand, CB2 receptor deletion exacerbated neuroinflammatory response in animal models of experimental autoimmune encephalomyelitis and cerebral ischemic/reperfusion injury (143–145). Thus, CB2 receptors seem to play a prominent role in inflammatory responses in the CNS. Its upregulation and activation may facilitate the downregulation and control of inflammatory processes (146). In agreement with this proposal, Ehrhart and colleagues showed that the CB2 receptor agonist JWH015 reduces IFN-γ-induced upregulation of CD40 expression in mouse microglia, which is involved in pathological activation of these cells (60).

In an animal model of Parkinson's disease, CB2 receptor activation reduced the neuroinflammatory process, brain-blood-barrier damage and T-cell infiltration, and increased nigrostriatal dopamine neuronal survival (147). *In vitro* studies demonstrated that the selective CB2 receptor agonists JWH133 and HU-308 reduced pro-inflammatory cytokines release in microglia culture (148, 149). The treatment with HU-308 decreased striatal neuroinflammation in a rodent model of L-dopa induced dyskinesia (150). This anti-inflammatory-like effect induced by the activation of CB2 receptors is also seen after a traumatic brain injury. The treatment with a selective CB2 receptor agonist decreased macrophage infiltration and pro-inflammatory cytokine expression, and increased M2 macrophage polarization (151). Other *in vivo* studies also demonstrated an anti-inflammatory effect of CB2 receptor activation in different animal models of neurodegenerative diseases (152–154).

In summary, some schizophrenia patients present marked microglia activation and increased levels of pro-inflammatory markers. The modulation of these changes as a strategy to treat this disorder seems promising (146). Given that the activation of CB2 receptors leads to the inhibition of microglial activation and the release of pro-inflammatory cytokines (65), these receptors have emerged as potential therapeutic targets (Figure 1).

CB2 receptors also seem to play a role in stress regulation. In mice, deletion of CB2 receptors increases stress responsivity (66) and stress exposure decreased hippocampus CB2 receptor expression (67). On the other hand, the genetically-induced overexpression of CB2 receptors produced anti-stress effect (68). In addition, the activation of CB2 receptors also induces anti-stress effects in rodents (65, 68, 155, 156). Exposure to stress, a well-known risk factor for the development of schizophrenia (157), increases microglia activation (158). Individuals at high risk of developing schizophrenia show increased responsivity to stress and are more likely to develop the disorder if they have decreased tolerance to stress (159). In animal models, stress relief during adolescence prevented the development of a schizophrenia phenotype at adulthood (160). Thus, the activation of CB2 receptors, due to its anti-stress effects (65, 68, 155, 156), may represent a strategy to prevent the transition from a high-risk state to full-scale schizophrenia. CB2 receptor may also be associated with anxiety and depression symptoms, which are clinical manifestations present in schizophrenia. A detailed discussion on this possibility was recently reviewed by Banaszkiewicz et al. (64).

### CB2 Receptors, Neurogenesis, and Synaptic Plasticity

Neuroplastic changes have also been associated with schizophrenia (161, 162). For instance, impaired adult hippocampal neurogenesis, which correlates with reduced cognitive function and affective symptoms (163), has been observed in patients with this disorder (164, 165). Corroborating these findings, *in vitro* models of hippocampal neurogenesis using fibroblasts-derived induced pluripotent stem cells (iPSCs) indicated that iPSCs from schizophrenia patients showed deficits in the generation of hippocampal granule neurons with lowered levels of adult neurogenesis-related genes (166). In addition, the lack of genes thought to regulate neurogenesis produced schizophrenia-like changes in mice (167).

Some authors suggest that impaired hippocampal neurogenesis may act as a susceptibility factor for schizophrenia development, then repairing and boosting neurogenesis may be beneficial (168). Preclinical studies have indicated a neuroprotective role of CB2 receptors against impaired adult hippocampal neurogenesis (169). Activation of these receptors also enhances the proliferation of embryonic and hippocampal neural progenitor cells and may increase neurogenesis (170, 171). Thus, CB2 receptor activation might improve cognitive deficits and affective schizophrenia symptoms through neuroprotective mechanisms against impaired neurogenesis. Corroborating this possibility, we have recently found that repeated CBD prevents synaptic remodeling and the decrease in hippocampal neurogenesis caused by chronic stress (108). In the hippocampus of stressed mice, CBD enhanced the branching and number of dendrite spines and increased the proliferation and migration of newborn granule cells. These effects were prevented by co-administration of the CB2 receptor antagonist AM630 (108). Similar effects have been described after clozapine administration (172). It remains to be further investigated if these CB2 receptor-mediated effects could play a role in schizophrenia by preventing stress-induced neuroplastic changes in susceptible individuals.

## Conclusion

Schizophrenia is a multifaceted disorder and is improbable that a single drug could adequately treat all its manifestations. So far, the available drug treatments have focused on trying to restore the hyperdopaminergic state seen in the disease. This approach is unmistakably insufficient in most patients and probably reflects the multifactorial pathophysiology of this disorder. A complementary approach would be to act on several targets involved in complex disorders. This approach could explain why clozapine, a multi-target compound, is still the more efficacious antipsychotic drug available (173).

After thirty years of their discovery, it has become clear that endocannabinoids play a fundamental modulatory role over not only several neurotransmitter systems and cellular processes such as immune responses that can play an important role in psychiatric disorders. As discussed above, the involvement of CB1 receptors in schizophrenia is still controversial. CB2 receptors, on the other hand, seem to modulate some of the critical processes associated with this disorder, meaning the dopaminergic, glutamatergic, and immune systems (see Figure 1). The potential of new therapies focused on these receptors needs to be further evaluated, particularly after long term administration in models based on neurodevelopmental disruption. In addition, given its role in regulating stress and neuroinflammation, the CB2 receptors may be more critical in early psychosis development than in chronic states.

## Author Contributions

All authors listed have made a substantial, direct and intellectual contribution to the work, and approved it for publication.

## Conflict of Interest

FSG is a co-inventor (Mechoulam R, JC, Guimaraes FS, AZ, JH, Breuer A) of the patent “Fluorinated CBD compounds, compositions and uses thereof. Pub. No.: WO/2014/108899. International Application No.: PCT/IL2014/050023” Def. US no. Reg. 62193296; 29/07/2015; INPI on 19/08/2015 (BR1120150164927). The University of São Paulo has licensed the patent to Phytecs Pharm (USP Resolution No. 15.1.130002.1.1). The University of São Paulo has an agreement with Prati-Donaduzzi (Toledo, Brazil) to “develop a pharmaceutical product containing synthetic cannabidiol and prove its safety and therapeutic efficacy in the treatment of epilepsy, schizophrenia, Parkinson's disease, and anxiety disorders.” The remaining authors declare that the research was conducted in the absence of any commercial or financial relationships that could be construed as a potential conflict of interest.

## References

[1] Kahn RS Sommer IE Murray RM Meyer-Lindenberg A Weinberger DR Cannon TD. Schizophrenia. Nat Rev Dis Prim. (2015) 1:15067. 10.1038/nrdp.2015.67 27189524

[2] Andreasen NC. Symptoms, signs, and diagnosis of schizophrenia. Lancet. (1995) 346:477–81. 10.1016/S0140-6736(95)91325-4 7637483

[3] Carbon M Correll CU. Thinking and acting beyond the positive: the role of the cognitive and negative symptoms in schizophrenia. CNS Spectr. (2014) 19:38–52. 10.1017/S1092852914000601 25403863

[4] Murray RM Lappin J Di Forti M. Schizophrenia: from developmental deviance to dopamine dysregulation. Eur Neuropsychopharmacol. (2008) 18(Suppl 3):S129–34. 10.1016/j.euroneuro.2008.04.002 18499406

[5] Howes OD McCutcheon R Owen MJ Murray RM. The role of genes, stress, and dopamine in the development of schizophrenia. Biol Psychiatry. (2017) 81:9–20. 10.1016/j.biopsych.2016.07.014 27720198 PMC5675052

[6] Nasrallah HA. Atypical antipsychotic-induced metabolic side effects: insights from receptor-binding profiles. Mol Psychiatry. (2008) 13:27–35. 10.1038/sj.mp.4002066 17848919

[7] Lieberman JA Stroup TS McEvoy JP Swartz MS Rosenheck RA Perkins DO. Effectiveness of antipsychotic drugs in patients with chronic schizophrenia. N Engl J Med. (2005) 353:1209–23. 10.1056/NEJMoa051688 16172203

[8] Solmi M Murru A Pacchiarotti I Undurraga J Veronese N Fornaro M. Safety, tolerability, and risks associated with first-and second-generation antipsychotics: a state-of-the-art clinical review. Ther Clin Risk Manag. (2017) 13:757–77. 10.2147/TCRM.S117321 28721057 PMC5499790

[9] Nuechterlein KH Barch DM Gold JM Goldberg TE Green MF Heaton RK. Identification of separable cognitive factors in schizophrenia. Schizophr Res. (2004) 72:29–39. 10.1016/j.schres.2004.09.007 15531405

[10] Keefe RSE Bilder RM Davis SM Harvey PD Palmer BW Gold JM. Neurocognitive effects of antipsychotic medications in patients with chronic schizophrenia in the CATIE trial. Arch Gen Psychiatry. (2007) 64:633–47. 10.1001/archpsyc.64.6.633 17548746

[11] Mintz J Kopelowicz A. CUtLASS confirms CATIE [2]. Arch Gen Psychiatry. (2007) 64:978–80. 10.1001/archpsyc.64.8.978-a 17679644

[12] Eggers AE. A serotonin hypothesis of schizophrenia. Med Hypotheses. (2013) 80:791–4. 10.1016/j.mehy.2013.03.013 23557849

[13] Glausier JR Lewis DA. GABA and schizophrenia: where we stand and where we need to go. Schizophr Res. (2017) 181:2–3. 10.1016/j.schres.2017.01.050 28179064 PMC5365350

[14] Stahl SM. Beyond the dopamine hypothesis of schizophrenia to three neural networks of psychosis: dopamine, serotonin, and glutamate. CNS Spectr. (2018) 23:187–91. 10.1017/S1092852918001013 29954475

[15] Uno Y Coyle JT. Glutamate hypothesis in schizophrenia. Psychiatry Clin Neurosci. (2019) 73:204–15. 10.1111/pcn.12823 30666759

[16] Volk DW Lewis DA. Insights into the pathophysiology of endocannabinoid signaling in schizophrenia. JAMA Psychiatry. (2019) 76:887–8. 10.1001/jamapsychiatry.2019.0844 31166594

[17] Lu HC Mackie K. An introduction to the endogenous cannabinoid system. Biol Psychiatry. (2016) 79:516–25. 10.1016/j.biopsych.2015.07.028 26698193 PMC4789136

[18] Maccarrone M. Endocannabinoids: friends and foes of reproduction. Prog Lipid Res. (2009) 48:344–54. 10.1016/j.plipres.2009.07.001 19602425

[19] Fezza F Bari M Florio R Talamonti E Feole M Maccarrone M. Endocannabinoids, related compounds and their metabolic routes. Molecules. (2014) 19:17078–106. 10.3390/molecules191117078 25347455 PMC6271436

[20] Chen DJ Gao M Gao FF Su QX Wu J. Brain cannabinoid receptor 2: expression, function and modulation. Acta Pharmacol Sin. (2017) 38:312–6. 10.1038/aps.2016.149 28065934 PMC5342669

[21] Cravatt BF Giang DK Mayfield SP Boger DL Lerner RA Gilula NB. Molecular characterization of an enzyme that degrades neuromodulatory fatty-acid amides. Nature. (1996) 384:83–7. 10.1038/384083a0 8900284

[22] Blankman JL Simon GM Cravatt BF. A Comprehensive profile of brain enzymes that hydrolyze the endocannabinoid 2-arachidonoylglycerol. Chem Biol. (2007) 14:1347–56. 10.1016/j.chembiol.2007.11.006 18096503 PMC2692834

[23] Howlett AC Barth F Bonner TI Cabral G Casellas P Devane WA. International Union of Pharmacology. XXVII. Classification of cannabinoid receptors. Pharmacol Rev. (2002) 54:161–202. 10.1124/pr.54.2.161 12037135

[24] Mackie K. Distribution of cannabinoid receptors in the central and peripheral nervous system. Handb Exp Pharmacol. (2005) 168:299–325. 10.1007/3-540-26573-2_10 16596779

[25] Fakhoury M. Role of the Endocannabinoid system in the pathophysiology of schizophrenia. Mol Neurobiol. (2017) 54:768–78. 10.1007/s12035-016-9697-5 26768595

[26] Pertwee RG. The diverse CB 1 and CB 2 receptor pharmacology of three plant cannabinoids: Δ 9-tetrahydrocannabinol, cannabidiol and Δ 9-tetrahydrocannabivarin. Br J Pharmacol. (2008) 153:199–215. 10.1038/sj.bjp.0707442 17828291 PMC2219532

[27] Renard J Krebs MO Le Pen G Jay TM. Long-term consequences of adolescent cannabinoid exposure in adult psychopathology. Front Neurosci. (2014) 8:361. 10.3389/fnins.2014.00361 25426017 PMC4226229

[28] Fernandez-Espejo E Viveros MP Núñez L Ellenbroek BA Rodriguez De Fonseca F. Role of cannabis and endocannabinoids in the genesis of schizophrenia. Psychopharmacology (Berl). (2009) 206:531–49. 10.1007/s00213-009-1612-6 19629449

[29] Silins E Horwood LJ Patton GC Fergusson DM Olsson CA Hutchinson DM. Young adult sequelae of adolescent cannabis use: an integrative analysis. Lancet Psychiatry. (2014) 1:286–93. 10.1016/S2215-0366(14)70307-4 26360862

[30] Ujike H Takaki M Nakata K Tanaka Y Takeda T Kodama M. CNR1, central cannabinoid receptor gene, associated with susceptibility to hebephrenic schizophrenia. Mol Psychiatry. (2002) 7:515–8. 10.1038/sj.mp.4001029 12082570

[31] Bae JS Kim JY Park BL Kim JH Kim B Park CS. Genetic association analysis of CNR1 and CNR2 polymorphisms with schizophrenia in a Korean population. Psychiatr Genet. (2014) 24:225–9. 10.1097/YPG.0000000000000047 25014618

[32] Hamdani N Tabeze JP Ramoz N Ades J Hamon M Sarfati Y. The CNR1 gene as a pharmacogenetic factor for antipsychotics rather than a susceptibility gene for schizophrenia. Eur Neuropsychopharmacol. (2008) 18:34–40. 10.1016/j.euroneuro.2007.05.005 17669634

[33] Guidali C Viganò D Petrosino S Zamberletti E Realini N Binelli G. Cannabinoid CB1 receptor antagonism prevents neurochemical and behavioural deficits induced by chronic phencyclidine. Int J Neuropsychopharmacol. (2011) 14:17–28. 10.1017/S1461145710000209 20196921

[34] Kruk-Slomka M Budzynska B Slomka T Banaszkiewicz I Biala G. The Influence of the CB1 receptor ligands on the schizophrenia-like effects in mice induced by MK-801. Neurotox Res. (2016) 30:658–76. 10.1007/s12640-016-9662-0 27577742 PMC5047950

[35] Dalton VS Long LE Weickert CS Zavitsanou K. Paranoid schizophrenia is characterized by increased CB 1 receptor binding in the dorsolateral prefrontal cortex. Neuropsychopharmacology. (2011) 36:1620–30. 10.1038/npp.2011.43 21471953 PMC3138655

[36] Giuffrida A Leweke FM Gerth CW Schreiber D Koethe D Faulhaber J. Cerebrospinal anandamide levels are elevated in acute schizophrenia and are inversely correlated with psychotic symptoms. Neuropsychopharmacology. (2004) 29:2108–14. 10.1038/sj.npp.1300558 15354183

[37] Koethe D Giuffrida A Schreiber D Hellmich M Schultze-Lutter F Ruhrmann S. Anandamide elevation in cerebrospinal fluid in initial prodromal states of psychosis. Br J Psychiatry. (2009) 194:371–2. 10.1192/bjp.bp.108.053843 19336792

[38] Bioque M García-Bueno B MacDowell KS Meseguer A Saiz PA Parellada M. Peripheral endocannabinoid system dysregulation in first-episode psychosis. Neuropsychopharmacology. (2013) 38:2568–77. 10.1038/npp.2013.165 23822951 PMC3828529

[39] Leweke FM Giuffrida A Wurster U Emrich HM Piomelli D. Elevated endogenous cannabinoids in schizophrenia. Neuroreport. (1999) 10:1665–9. 10.1097/00001756-199906030-00008 10501554

[40] De Marchi N De Petrocellis L Orlando P Daniele F Fezza F Di Marzo V. Endocannabinoid signalling in the blood of patients with schizophrenia. Lipids Health Dis. (2003) 2:5. 10.1186/1476-511X-2-5 12969514 PMC194767

[41] Zuardi AW Guimarães FS Hallak JEC Crippa JAS. Is the highest density of CB1 receptors in paranoid schizophrenia a correlate of endocannabinoid system functioning? Expert Rev Neurother. (2011) 11:1111–4. 10.1586/ern.11.89 21797651

[42] Munro S Thomas KL Abu-Shaar M. Molecular characterization of a peripheral receptor for cannabinoids. Nature. (1993) 365:61–5. 10.1038/365061a0 7689702

[43] Ashton J Glass M. The cannabinoid CB2 receptor as a target for inflammation-dependent neurodegeneration. Curr Neuropharmacol. (2007) 5:73–80. 10.2174/157015907780866884 18615177 PMC2435344

[44] Cabral GA; Griffin-Thomas L. Emerging role of the CB2 cannabinoid receptor in immune regulation and therapeutic prospects. Expert Rev Mol Med. (2009) 11:e3. 10.1017/S1462399409000957 19152719 PMC2768535

[45] Turcotte C Blanchet MR Laviolette M Flamand N. The CB2 receptor and its role as a regulator of inflammation. Cell Mol Life Sci. (2016) 73:4449–70. 10.1007/s00018-016-2300-4 27402121 PMC5075023

[46] Bie B Wu J Foss JF Naguib M. An overview of the cannabinoid type 2 receptor system and its therapeutic potential. Curr Opin Anaesthesiol. (2018) 31:407–14. 10.1097/ACO.0000000000000616 29794855 PMC6035094

[47] SvíŽenská I Dubový P Šulcová A. Cannabinoid receptors 1 and 2 (CB1 and CB2), their distribution, ligands and functional involvement in nervous system structures - a short review. Pharmacol Biochem Behav. (2008) 90:501–11. 10.1016/j.pbb.2008.05.010 18584858

[48] Atwood BK MacKie K. CB 2: a cannabinoid receptor with an identity crisis. Br J Pharmacol. (2010) 160:467–79. 10.1111/j.1476-5381.2010.00729.x 20590558 PMC2931549

[49] Gong JP Onaivi ES Ishiguro H Liu QR Tagliaferro PA Brusco A. Cannabinoid CB2 receptors: immunohistochemical localization in rat brain. Brain Res. (2006) 1071:10–23. 10.1016/j.brainres.2005.11.035 16472786

[50] Sickle MD Van Duncan M Kingsley PJ Mouihate A Urbani P Mackie K. Identification and functional characterization of brainstem cannabinoid CB2 receptor. Science. (2005) 310:329–32. 10.1126/science.1115740 16224028

[51] Racz I Nadal X Alferink J Baños JE Rehnelt J Martín M. Crucial role of CB2 cannabinoid receptor in the regulation of central immune responses during neuropathic pain. J Neurosci. (2008) 28:12125–35. 10.1523/JNEUROSCI.3400-08.2008 19005077 PMC3844839

[52] Onaivi ES Ishiguro H Gong JP Patel S Meozzi PA Myers L. Functional expression of brain neuronal CB2 cannabinoid receptors are involved in the effects of drugs of abuse and in depression. Ann N Y Acad Sci. (2008) 1139:43449. 10.1196/annals.1432.036 18991891 PMC3922202

[53] Onaivi ES Ishiguro H Gu S Liu QR. CNS effects of CB2 cannabinoid receptors: Beyond neuro-immuno-cannabinoid activity. J Psychopharmacol. (2012) 26:92–103. 10.1177/0269881111400652 21447538 PMC3388033

[54] Sánchez-Zavaleta R Cortés H Avalos-Fuentes JA García U Segovia-Vila J Erlij D. Presynaptic cannabinoid CB2 receptors modulate [3 H]-Glutamate release at subthalamo-nigral terminals of the rat. Synapse. (2018) 72:52–5. 10.1002/syn.22061 30022523

[55] Miller LK Devi LA. The highs and lows of cannabinoid receptor expression in disease: Mechanisms and their therapeutic implications. Pharmacol Rev. (2011) 63:461–70. 10.1124/pr.110.003491 21752875 PMC3141881

[56] Benito C Tolón RM Pazos MR Núñez E Castillo AI Romero J. Cannabinoid CB2 receptors in human brain inflammation. Br J Pharmacol. (2008) 153:277–85. 10.1038/sj.bjp.0707505 17934510 PMC2219537

[57] Lisboa SF Gomes F V. Guimaraes FS Campos AC. Microglial cells as a link between cannabinoids and the immune hypothesis of psychiatric disorders. Front Neurol. (2016) 7:5. 10.3389/fneur.2016.00005 26858686 PMC4729885

[58] Walter L Franklin A Witting A Wade C Xie Y Kunos G. Nonpsychotropic cannabinoid receptors regulate microglial cell migration. J Neurosci. (2003) 23:1398–405. 10.1523/JNEUROSCI.23-04-01398.2003 12598628 PMC6742252

[59] Carrier EJ Kearn CS Barkmeier AJ Breese NM Yang W Nithipatikom K. Cultured rat microglial cells synthesize the endocannabinoid 2-arachidonylglycerol, which increases proliferation via a CB2 receptor-dependent mechanism. Mol Pharmacol. (2004) 65:999–1007. 10.1124/mol.65.4.999 15044630

[60] Ehrhart J Obregon D Mori T Hou H Sun N Bai Y. Stimulation of cannabinoid receptor 2 (CB2) suppresses microglial activation. J Neuroinflammation. (2005) 2:29. 10.1186/1742-2094-2-29 16343349 PMC1352348

[61] Fernández-Ruiz J Pazos MR García-Arencibia M Sagredo O Ramos JA. Role of CB2 receptors in neuroprotective effects of cannabinoids. Mol Cell Endocrinol. (2008) 286(1-2 Suppl 1):S91–6. 10.1016/j.mce.2008.01.001 18291574

[62] Mechoulam R Parker LA. The endocannabinoid system and the brain. Annu Rev Psychol. (2013) 64:21–47. 10.1146/annurev-psych-113011-143739 22804774

[63] Cassano T Calcagnini S Pace L Marco F De Romano A Gaetani S. Cannabinoid receptor 2 signaling in neurodegenerative disorders: from pathogenesis to a promising therapeutic target. Front Neurosci. (2017) 11:30. 10.3389/fnins.2017.00030 28210207 PMC5288380

[64] Banaszkiewicz I Biala G Kruk-Slomka M. Contribution of CB2 receptors in schizophrenia-related symptoms in various animal models: short review. Neurosci Biobehav Rev. (2020) 114:158–71. 10.1016/j.neubiorev.2020.04.020 32437746

[65] Roche M Finn DP. Brain CB2 receptors: implications for neuropsychiatric disorders. Pharmaceuticals. (2010) 3:2517–33. 10.3390/ph3082517 27713365 PMC4033937

[66] Ortega-Alvaro A Aracil-Fernández A García-Gutiérrez MS Navarrete F Manzanares J. Deletion of CB2 cannabinoid receptor induces schizophrenia-related behaviors in mice. Neuropsychopharmacology. (2011) 36:1489–504. 10.1038/npp.2011.34 21430651 PMC3096817

[67] Ishiguro H Horiuchi Y Tabata K Liu QR Arinami T Onaivi ES. Cannabinoid CB2 receptor gene and environmental interaction in the development of psychiatric disorders. Molecules. (2018) 23:1836. 10.3390/molecules23081836 30042304 PMC6114128

[68] García-Gutiérrez MS Pérez-Ortiz JM Gutiérrez-Adán A Manzanares J. Depression-resistant endophenotype in mice overexpressing cannabinoid CB 2 receptors. Br J Pharmacol. (2010) 160:1773–84. 10.1111/j.1476-5381.2010.00819.x 20649579 PMC2936848

[69] Khandaker GM Cousins L Deakin J Lennox BR Yolken R Jones PB. Inflammation and immunity in schizophrenia: implications for pathophysiology and treatment. Lancet Psychiatry. (2015) 2:258–70. 10.1016/S2215-0366(14)00122-9 26359903 PMC4595998

[70] Leonard BE. Inflammation and depression: a causal or coincidental link to the pathophysiology? Acta Neuropsychiatr. (2018) 30:1–16. 10.1017/neu.2016.69 28112061

[71] Ishiguro H Horiuchi Y Ishikawa M Koga M Imai K Suzuki Y. Brain cannabinoid CB2 receptor in schizophrenia. Biol Psychiatry. (2010) 67:974–82. 10.1016/j.biopsych.2009.09.024 19931854

[72] Legge SE Jones HJ Kendall KM Pardiñas AF Menzies G Bracher-Smith M. Association of genetic liability to psychotic experiences with neuropsychotic disorders and traits. JAMA Psychiatry. (2019) 76:1256–65. 10.1001/jamapsychiatry.2019.2508 31553412 PMC6764002

[73] Grace AA Gomes FV. The circuitry of dopamine system regulation and its disruption in schizophrenia: insights into treatment and prevention. Schizophr Bull. (2019) 45:148–57. 10.1093/schbul/sbx199 29385549 PMC6293217

[74] Grace AA. Dopamine system dysregulation and the pathophysiology of schizophrenia: insights from the methylazoxymethanol acetate model. Biol Psychiatry. (2017) 81:5–8. 10.1016/j.biopsych.2015.11.007 26705848 PMC4870144

[75] Fiorillo CD Williams JT. Glutamate mediates an inhibitory postsynaptic potential in dopamine neurons. Nature. (1998) 394:78–82. 10.1038/27919 9665131

[76] Paladini CA Tepper JM. Neurophysiology of substantia Nigra dopamine neurons: Modulation by GABA and glutamate. Handb Behav Neurosci. (2016) 24:335–60. 10.1016/B978-0-12-802206-1.00017-9

[77] Beckstead MJ Grandy DK Wickman K Williams JT. Vesicular dopamine release elicits an inhibitory postsynaptic current in midbrain dopamine neurons. Neuron. (2004) 42:939–46. 10.1016/j.neuron.2004.05.019 15207238

[78] Canseco-Alba A Schanz N Sanabria B Zhao J Lin Z Liu QR. Behavioral effects of psychostimulants in mutant mice with cell-type specific deletion of CB2 cannabinoid receptors in dopamine neurons. Behav Brain Res. (2019) 360:286–97. 10.1016/j.bbr.2018.11.043 30508607 PMC6327973

[79] Xi ZX Peng XQ Li X Song R Zhang HY Liu QR. Brain cannabinoid CB2 receptors modulate cocaine's actions in mice. Nat Neurosci. (2011) 14:1160–6. 10.1038/nn.2874 21785434 PMC3164946

[80] Manzanares J Cabañero D Puente N García-Gutiérrez MS Grandes P Maldonado R. Role of the endocannabinoid system in drug addiction. Biochem Pharmacol. (2018) 157:108–21. 10.1016/j.bcp.2018.09.013 30217570

[81] Diana M Melis M Gessa GL. Increase in meso-prefrontal dopaminergic activity after stimulation of CB1 receptors by cannabinoids. Eur J Neurosci. (1998) 10:2825–30. 10.1111/j.1460-9568.1998.00292.x 9758152

[82] Riegel AC Lupica CR. Independent presynaptic and postsynaptic mechanisms regulate endocannabinoid signaling at multiple synapses in the ventral tegmental area. J Neurosci. (2004) 24:11070–8. 10.1523/JNEUROSCI.3695-04.2004 15590923 PMC4857882

[83] Ward SJ Rosenberg M Dykstra LA Walker EA. The CB1 antagonist rimonabant (SR141716) blocks cue-induced reinstatement of cocaine seeking and other context and extinction phenomena predictive of relapse. Drug Alcohol Depend. (2009) 105:248–55. 10.1016/j.drugalcdep.2009.07.002 19679410 PMC2763982

[84] Yu LL Zhou SJ Wang XY Liu JF Xue YX Jiang W. Effects of cannabinoid CB1 receptor antagonist rimonabant on acquisition and reinstatement of psychostimulant reward memory in mice. Behav Brain Res. (2011) 217:111–6. 10.1016/j.bbr.2010.10.008 20937331

[85] Christensen R Kristensen PK Bartels EM Bliddal H Astrup A. Efficacy and safety of the weight-loss drug rimonabant: a meta-analysis of randomised trials. Lancet. (2007) 370:1706–13. 10.1016/S0140-6736(07)61721-8 18022033

[86] Filip M Papla I Nowak E Przegaliński E. Blocking impact of clozapine on cocaine locomotor and sensitizing effects in rats. Pol J Pharmacol. (2003) 55:1125–30. 14730110

[87] Delis F Polissidis A Poulia N Justinova Z Nomikos GG Goldberg SR. Attenuation of cocaine-induced conditioned place preference and motor activity via cannabinoid CB2 receptor agonism and cb1 receptor antagonism in rats. Int J Neuropsychopharmacol. (2017) 20:269–78. 10.1093/ijnp/pyw102 27994006 PMC5408977

[88] Lopes JB Bastos JR Costa RB Aguiar DC Moreira FA. The roles of cannabinoid CB1 and CB2 receptors in cocaine-induced behavioral sensitization and conditioned place preference in mice. Psychopharmacology (Berl). (2020) 237:385–94. 10.1007/s00213-019-05370-5 31667531

[89] Aracil-Fernández A Trigo JM García-Gutiérrez MS Ortega-Álvaro A Ternianov A Navarro D. Decreased cocaine motor sensitization and self-administration in mice overexpressing cannabinoid CB2receptors. Neuropsychopharmacology. (2012) 37:1749–63. 10.1038/npp.2012.22 22414816 PMC3358745

[90] Zhang HY Gao M Liu QR Bi GH Li X Yang HJ. Cannabinoid CB2 receptors modulate midbrain dopamine neuronal activity and dopamine-related behavior in mice. Proc Natl Acad Sci U S A. (2014) 111:E5007–15. 10.1073/pnas.1413210111 25368177 PMC4246322

[91] Zhang HY Gao M Shen H Bi GH Yang HJ Liu QR. Expression of functional cannabinoid CB2 receptor in VTA dopamine neurons in rats. Addict Biol. (2017) 22:752–65. 10.1111/adb.12367 26833913 PMC4969232

[92] Liu QR Canseco-Alba A Zhang HY Tagliaferro P Chung M Dennis E. Cannabinoid type 2 receptors in dopamine neurons inhibits psychomotor behaviors, alters anxiety, depression and alcohol preference. Sci Rep. (2017) 7:17410. 10.1038/s41598-017-17796-y 29234141 PMC5727179

[93] Ma Z Gao F Larsen B Gao M Luo Z Chen D. Mechanisms of cannabinoid CB 2 receptor-mediated reduction of dopamine neuronal excitability in mouse ventral tegmental area. EBioMedicine. (2019) 42:225–37. 10.1016/j.ebiom.2019.03.040 30952618 PMC6491419

[94] Foster DJ Wilson JM Remke DH Mahmood MS Uddin MJ Wess J. Antipsychotic-like effects of M4 positive allosteric modulators are mediated by CB2 receptor-dependent inhibition of dopamine release. Neuron. (2016) 91:1244–52. 10.1016/j.neuron.2016.08.017 27618677 PMC5033724

[95] Krystal JH. Subanesthetic effects of the noncompetitive NMDA antagonist, ketamine, in humans. Arch Gen Psychiatry. (1994) 51:199–214. 10.1001/archpsyc.1994.03950030035004 8122957

[96] Krystal JH Perry EB Gueorguieva R Belger A Madonick SH Abi-Dargham A. Comparative and interactive human psychopharmacologic effects of ketamine and amphetamine: implications for glutamatergic and dopaminergic model psychoses and cognitive function. Arch Gen Psychiatry. (2005) 62:985–94. 10.1001/archpsyc.62.9.985 16143730

[97] Bubeníková-Valešová V Horáček J Vrajová M Höschl C. Models of schizophrenia in humans and animals based on inhibition of NMDA receptors. Neurosci Biobehav Rev. (2008) 32:1014–23. 10.1016/j.neubiorev.2008.03.012 18471877

[98] Gonzalez-Burgos G Lewis DA. NMDA receptor hypofunction, parvalbumin-positive neurons, and cortical gamma oscillations in schizophrenia. Schizophr Bull. (2012) 38:950–7. 10.1093/schbul/sbs010 22355184 PMC3446219

[99] Hudson MR Sokolenko E O'Brien TJ Jones NC. NMDA receptors on parvalbumin-positive interneurons and pyramidal neurons both contribute to MK-801 induced gamma oscillatory disturbances: complex relationships with behaviour. Neurobiol Dis. (2020) 134:104625. 10.1016/j.nbd.2019.104625 31786371

[100] Kaar SJ Angelescu I Marques TR Howes OD. Pre-frontal parvalbumin interneurons in schizophrenia: a meta-analysis of post-mortem studies. J Neural Transm (Vienna). (2019) 126:1637–51. 10.1007/s00702-019-02080-2 31529297 PMC6856257

[101] Steullet P Cabungcal JH Coyle J Didriksen M Gill K Grace AA. Oxidative stress-driven parvalbumin interneuron impairment as a common mechanism in models of schizophrenia. Mol Psychiatry. (2017) 22:936–43. 10.1038/mp.2017.47 28322275 PMC5491690

[102] Gomes F V. Issy AC Ferreira FR Viveros MP Del Bel EA Guimaraes FS. Cannabidiol attenuates sensorimotor gating disruption and molecular changes induced by chronic antagonism of NMDA receptors in Mice. Int J Neuropsychopharmacol. (2015) 18:1–10. 10.1093/ijnp/pyu041 25618402 PMC4376539

[103] Behrens MM Ali SS Dao DN Lucero J Shekhtman G Quick KL. Ketamine-induced loss of phenotype of fast-spiking interneurons is mediated by NADPH-oxidase. Science. (2007) 318:1645–7. 10.1126/science.1148045 18063801

[104] Khella R Short JL Malone DT. CB2 receptor agonism reverses MK-801-induced disruptions of prepulse inhibition in mice. Psychopharmacology (Berl). (2014) 231:3071–87. 10.1007/s00213-014-3481-x 24705902

[105] Kruk-Slomka M Banaszkiewicz I Biala G. The impact of CB2 receptor ligands on the MK-801-induced hyperactivity in mice. Neurotox Res. (2017) 31:410–20. 10.1007/s12640-017-9702-4 28138895 PMC5360834

[106] Zuardi AW Crippa JAS Hallak JEC Bhattacharyya S Atakan Z Martin-Santos R. A critical review of the antipsychotic effects of cannabidiol: 30 years of a translational investigation. Curr Pharm Des. (2012) 18:5131–40. 10.2174/138161212802884681 22716160

[107] Campos AC Moreira FA Gomes FV del Bel EA Guimarães FS. Multiple mechanisms involved in the large-spectrum therapeutic potential of cannabidiol in psychiatric disorders. Philos Trans R Soc B Biol Sci. (2012) 367:3364–78. 10.1098/rstb.2011.0389 23108553 PMC3481531

[108] Fogaça M V Campos AC Coelho LD Duman RS Guimarães FS. The anxiolytic effects of cannabidiol in chronically stressed mice are mediated by the endocannabinoid system: role of neurogenesis and dendritic remodeling. Neuropharmacology. (2018) 135:22–33. 10.1016/j.neuropharm.2018.03.001 29510186

[109] Galaj E Bi GH Yang HJ Xi ZX. Cannabidiol attenuates the rewarding effects of cocaine in rats by CB2, 5-HT1A and TRPV1 receptor mechanisms. Neuropharmacology. (2020) 167:107740. 10.1016/j.neuropharm.2019.107740 31437433 PMC7493134

[110] Rodrigues da Silva N Gomes FV Sonego AB Silva NR da Guimarães FS. Cannabidiol attenuates behavioral changes in a rodent model of schizophrenia through 5-HT1A, but not CB1 and CB2 receptors. Pharmacol Res. (2020) 156:104749. 10.1016/j.phrs.2020.104749 32151683

[111] Gomes F V Llorente R Del Bel EA Viveros MP López-Gallardo M Guimarães FS. Decreased glial reactivity could be involved in the antipsychotic-like effect of cannabidiol. Schizophr Res. (2015) 164:155–63. 10.1016/j.schres.2015.01.015 25680767

[112] Drzyzga Ł Obuchowicz E Marcinowska A Herman ZS. Cytokines in schizophrenia and the effects of antipsychotic drugs. Brain Behav Immun. (2006) 20:532–45. 10.1016/j.bbi.2006.02.002 16580814

[113] Taga T Fukuda S. Role of IL-6 in the neural stem cell differentiation. Clin Rev Allergy Immunol. (2005) 28:249–56. 10.1385/CRIAI:28:3:249 16129909

[114] Petitto JM McCarthy DB Rinker CM Huang Z Getty T. Modulation of behavioral and neurochemical measures of forebrain dopamine function in mice by species-specific interleukin-2. J Neuroimmunol. (1997) 73:183–90. 10.1016/S0165-5728(96)00196-8 9058775

[115] Wilson CJ Finch CE Cohen HJ. Cytokines and cognition - the case for a head-to-toe inflammatory paradigm. J Am Geriatr Soc. (2002) 50:2041–56. 10.1046/j.1532-5415.2002.50619.x 12473019

[116] Licinio J. Central nervous system cytokines and their relevance for neurotoxicity and apoptosis. J Neural Transm Suppl. (1997) 49:169–75. 10.1007/978-3-7091-6844-8_18 9266426

[117] Rothwell NJ Relton JK. Involvement of cytokines in acute neurodegeneration in the CNS. Neurosci Biobehav Rev. (1993) 17:217–27. 10.1016/S0149-7634(05)80152-6 8515904

[118] Wolf SA Boddeke HWGM Kettenmann H. Microglia in physiology and disease. Annu Rev Physiol. (2017) 79:619–43. 10.1146/annurev-physiol-022516-034406 27959620

[119] Sellgren CM Gracias J Watmuff B Biag JD Thanos JM Whittredge PB. Increased synapse elimination by microglia in schizophrenia patient-derived models of synaptic pruning. Nat Neurosci. (2019) 22:374–85. 10.1038/s41593-018-0334-7 30718903 PMC6410571

[120] Perkins DO Jeffries CD Do KQ. Potential roles of redox dysregulation in the development of schizophrenia. Biol Psychiatry. (2020) 88:326–36. 10.1016/j.biopsych.2020.03.016 32560962 PMC7395886

[121] Lin A Kenis G Bignotti S Tura GJB De Jong R Bosmans E. The inflammatory response system in treatment-resistant schizophrenia: Increased serum interleukin-6. Schizophr Res. (1998) 32:9–15. 10.1016/S0920-9964(98)00034-6 9690329

[122] Zhang XY Zhou DF Zhang PY Wu GY Cao LY Shen YC. Elevated interleukin-2, interleukin-6 and interleukin-8 serum levels in neuroleptic-free schizophrenia : Association with psychopathology. Schizophr Res. (2002) 57:247–58. 10.1016/S0920-9964(01)00296-1 12223256

[123] Söderlund J Schröder J Nordin C Samuelsson M Walther-Jallow L Karlsson H. Activation of brain interleukin-1? in schizophrenia. Mol Psychiatry. (2009) 14:1069–71. 10.1038/mp.2009.52 19920835 PMC2848473

[124] Ashdown H Dumont Y Ng M Poole S Boksa P Luheshi GN. The role of cytokines in mediating effects of prenatal infection on the fetus: Implications for schizophrenia. Mol Psychiatry. (2006) 11:47–55. 10.1038/sj.mp.4001748 16189509

[125] Bilbo SD Schwarz JM. Early-life programming of later-life brain and behavior: a critical role for the immune system. Front Behav Neurosci. (2009) 3:14. 10.3389/neuro.08.014.2009 19738918 PMC2737431

[126] Bland ST Beckley JT Young S Tsang V Watkins LR Maier SF. Enduring consequences of early-life infection on glial and neural cell genesis within cognitive regions of the brain. Brain Behav Immun. (2010) 24:329–38. 10.1016/j.bbi.2009.09.012 19782746 PMC2826544

[127] Van Kesteren CFMG Gremmels H De Witte LD Hol EM Van Gool AR Falkai PG. Immune involvement in the pathogenesis of schizophrenia: a meta-analysis on postmortem brain studies. Transl Psychiatry. (2017) 7:e1075. 10.1038/tp.2017.4 28350400 PMC5404615

[128] van Berckel BN Bossong MG Boellaard R Kloet R Schuitemaker A Caspers E. Microglia activation in recent-onset schizophrenia: a quantitative (R)-[11C]PK11195 positron emission tomography study. Biol Psychiatry. (2008) 64:820–2. 10.1016/j.biopsych.2008.04.025 18534557

[129] Doorduin J De Vries EFJ Willemsen ATM De Groot JC Dierckx RA Klein HC. Neuroinflammation in schizophrenia-related psychosis: a PET study. J Nucl Med. (2009) 50:1801–7. 10.2967/jnumed.109.066647 19837763

[130] Takano A Arakawa R Ito H Tateno A Takahashi H Matsumoto R. Peripheral benzodiazepine receptors in patients with chronic schizophrenia: a PET study with [11C]DAA1106. Int J Neuropsychopharmacol. (2010) 13:943–50. 10.1017/S1461145710000313 20350336

[131] Miyaoka T Yasukawa R Yasuda H Hayashida M Inagaki T Horiguchi J. Possible antipsychotic effects of minocycline in patients with schizophrenia. Prog Neuropsychopharmacol Biol Psychiatry. (2007) 31:304–7. 10.1016/j.pnpbp.2006.08.013 17030375

[132] Fujita Y Ishima T Kunitachi S Hagiwara H Zhang L Iyo M. Phencyclidine-induced cognitive deficits in mice are improved by subsequent subchronic administration of the antibiotic drug minocycline. Prog Neuropsychopharmacol Biol Psychiatry. (2008) 32:336–9. 10.1016/j.pnpbp.2007.08.031 17884273

[133] Levkovitz Y Mendlovich S Riwkes S Braw Y Levkovitch-Verbin H Gal G. A double-blind, randomized study of minocycline for the treatment of negative and cognitive symptoms in early-phase schizophrenia. J Clin Psychiatry. (2010) 71:138–49. 10.4088/JCP.08m04666yel 19895780

[134] Zhang L Zheng H Wu R Zhu F Kosten TR Zhang XY. Minocycline adjunctive treatment to risperidone for negative symptoms in schizophrenia: Association with pro-inflammatory cytokine levels. Prog Neuropsychopharmacol Biol Psychiatry. (2018) 85:69–76. 10.1016/j.pnpbp.2018.04.004 29678772

[135] Deakin B Suckling J Barnes TRE Byrne K Chaudhry IB Dazzan P. The benefit of minocycline on negative symptoms of schizophrenia in patients with recent-onset psychosis (BeneMin): a randomised, double-blind, placebo-controlled trial. Lancet Psychiatry. (2018) 5:885–94. 10.1016/S2215-0366(18)30345-6 30322824 PMC6206257

[136] Müller N Riedel M Schwarz MJ Engel RR. Clinical effects of COX-2 inhibitors on cognition in schizophrenia. Eur Arch Psychiatry Clin Neurosci. (2005) 255:149–51. 10.1007/s00406-004-0548-4 15549344

[137] Akhondzadeh S Tabatabaee M Amini H Ahmadi Abhari SA Abbasi SH Behnam B. Celecoxib as adjunctive therapy in schizophrenia: a double-blind, randomized and placebo-controlled trial. Schizophr Res. (2007) 90:179–85. 10.1016/j.schres.2006.11.016 17208413

[138] Carlisle SJ Marciano-Cabral F Staab A Ludwick C Cabral GA. Differential expression of the CB2 cannabinoid receptor by rodent macrophages and macrophage-like cells in relation to cell activation. Int Immunopharmacol. (2002) 2:69–82. 10.1016/S1567-5769(01)00147-3 11789671

[139] Maresz K Carrier EJ Ponomarev ED Hillard CJ Dittel BN. Modulation of the cannabinoid CB2 receptor in microglial cells in response to inflammatory stimuli. J Neurochem. (2005) 95:437–45. 10.1111/j.1471-4159.2005.03380.x 16086683

[140] Fernández-Ruiz J Romero J Velasco G Tolón RM Ramos JA Guzmán M. Cannabinoid CB2 receptor: a new target for controlling neural cell survival? Trends Pharmacol Sci. (2007) 28:39–45. 10.1016/j.tips.2006.11.001 17141334

[141] Orihuela R McPherson CA Harry GJ. Microglial M1/M2 polarization and metabolic states. Br J Pharmacol. (2016) 173:649–65. 10.1111/bph.13139 25800044 PMC4742299

[142] Mecha M Feliú A Carrillo-Salinas FJ Rueda-Zubiaurre A Ortega-Gutiérrez S de Sola RG. Endocannabinoids drive the acquisition of an alternative phenotype in microglia. Brain Behav Immun. (2015) 49:233–45. 10.1016/j.bbi.2015.06.002 26086345

[143] Palazuelos J Davoust N Julien B Hatterer E Aguado T Mechoulam R. The CB2 cannabinoid receptor controls myeloid progenitor trafficking: involvement in the pathogenesis of an animal model of multiple sclerosis. J Biol Chem. (2008) 283:13320–9. 10.1074/jbc.M707960200 18334483

[144] Zhang M Adler MW Abood ME Ganea D Jallo J Tuma RF. CB2 receptor activation attenuates microcirculatory dysfunction during cerebral ischemic/reperfusion injury. Microvasc Res. (2009) 78:86–94. 10.1016/j.mvr.2009.03.005 19332079 PMC3319431

[145] Zarruk JG Fernández-López D García-Yébenes I García-Gutiérrez MS Vivancos J Nombela F. Cannabinoid type 2 receptor activation downregulates stroke-induced classic and alternative brain macrophage/microglial activation concomitant to neuroprotection. Stroke. (2012) 43:211–9. 10.1161/STROKEAHA.111.631044 22020035

[146] Monji A Kato TA Mizoguchi Y Horikawa H Seki Y Kasai M. Neuroinflammation in schizophrenia especially focused on the role of microglia. Prog Neuropsychopharmacol Biol Psychiatry. (2013) 42:115–21. 10.1016/j.pnpbp.2011.12.002 22192886

[147] Chung YC Shin WH Baek JY Cho EJ Baik HH Kim SR. CB2 receptor activation prevents glial-derived neurotoxic mediator production, BBB leakage and peripheral immune cell infiltration and rescues dopamine neurons in the MPTP model of Parkinson's disease. Exp Mol Med. (2016) 48:e205. 10.1038/emm.2015.100 27534533 PMC4892852

[148] Ramírez BG Blázquez C Gómez Del Pulgar T Guzmán M De Ceballos ML. Prevention of Alzheimer's disease pathology by cannabinoids: Neuroprotection mediated by blockade of microglial activation. J Neurosci. (2005) 25:1904–13. 10.1523/JNEUROSCI.4540-04.2005 15728830 PMC6726060

[149] Martín-Moreno AM Reigada D Ramírez BG Mechoulam R Innamorato N Cuadrado A. Cannabidiol and other cannabinoids reduce microglial activation *in vitro* and *in vivo*: relevance to Alzheimer's disease. Mol Pharmacol. (2011) 79:964–73. 10.1124/mol.111.071290 21350020 PMC3102548

[150] Rentsch P Stayte S Egan T Clark I Vissel B. Targeting the cannabinoid receptor CB2 in a mouse model of l-dopa induced dyskinesia. Neurobiol Dis. (2020) 134:104646. 10.1016/j.nbd.2019.104646 31669673

[151] Braun M Khan ZT Khan MB Kumar M Ward A Achyut BR. Selective activation of cannabinoid receptor-2 reduces neuroinflammation after traumatic brain injury via alternative macrophage polarization. Brain Behav Immun. (2018) 68:224–37. 10.1016/j.bbi.2017.10.021 29079445 PMC5767553

[152] Gómez-Gálvez Y Palomo-Garo C Fernández-Ruiz J García C. Potential of the cannabinoid CB2 receptor as a pharmacological target against inflammation in Parkinson's disease. Prog Neuropsychopharmacol Biol Psychiatry. (2016) 64:200–8. 10.1016/j.pnpbp.2015.03.017 25863279

[153] Sagredo O González S Aroyo I Pazos MR Benito C Lastres-Becker I. Cannabinoid CB2 receptor agonists protect the striatum against malonate toxicity: relevance for Huntington's disease. Glia. (2009) 57:1154–67. 10.1002/glia.20838 19115380 PMC2706932

[154] Palazuelos J Aguado T Pazos MR Julien B Carrasco C Resel E. Microglial CB2 cannabinoid receptors are neuroprotective in Huntington's disease excitotoxicity. Brain. (2009) 132:3152–64. 10.1093/brain/awp239 19805493

[155] Hu B Doods H Treede RD Ceci A. Depression-like behaviour in rats with mononeuropathy is reduced by the CB2-selective agonist GW405833. Pain. (2009) 143:206–12. 10.1016/j.pain.2009.02.018 19345493

[156] Kruk-Slomka M Michalak A Biala G. Antidepressant-like effects of the cannabinoid receptor ligands in the forced swimming test in mice: mechanism of action and possible interactions with cholinergic system. Behav Brain Res. (2015) 284:24–36. 10.1016/j.bbr.2015.01.051 25660201

[157] Gomes F V Zhu X Grace AA. Stress during critical periods of development and risk for schizophrenia. Schizophr Res. (2019) 213:107–13. 10.1016/j.schres.2019.01.030 30711313 PMC6667322

[158] Walker F Nilsson M Jones K. Acute and chronic stress-induced disturbances of microglial plasticity, phenotype and function. Curr Drug Targets. (2013) 14:1262–76. 10.2174/13894501113149990208 24020974 PMC3788324

[159] Pruessner M Iyer SN Faridi K Joober R Malla AK. Stress and protective factors in individuals at ultra-high risk for psychosis, first episode psychosis and healthy controls. Schizophr Res. (2011) 129:29–35. 10.1016/j.schres.2011.03.022 21497058

[160] Du Y Grace AA. Peripubertal diazepam administration prevents the emergence of dopamine system hyperresponsivity in the MAM developmental disruption model of schizophrenia. Neuropsychopharmacology. (2013) 38:1881–8. 10.1038/npp.2013.101 23612434 PMC3746684

[161] Yun S Reynolds RP Masiulis I Eisch AJ. Re-evaluating the link between neuropsychiatric disorders and dysregulated adult neurogenesis. Nat Med. (2016) 22:1239–47. 10.1038/nm.4218 27783068 PMC5791154

[162] Flores G Morales-Medina JC Diaz A. Neuronal and brain morphological changes in animal models of schizophrenia. Behav Brain Res. (2016) 301:190–203. 10.1016/j.bbr.2015.12.034 26738967

[163] Kang E Wen Z Song H Christian KM Ming GL. Adult neurogenesis and psychiatric disorders. Cold Spring Harb Perspect Biol. (2016) 8:a019026. 10.1101/cshperspect.a019026 26801682 PMC5008067

[164] Walton NM Zhou Y Kogan JH Shin R Webster M Gross AK. Detection of an immature dentate gyrus feature in human schizophrenia/bipolar patients. Transl Psychiatry. (2012) 2:e135. 10.1038/tp.2012.56 22781168 PMC3410619

[165] Allen KM Fung SJ Shannon Weickert C. Cell proliferation is reduced in the hippocampus in schizophrenia. Aust N Z J Psychiatry. (2016) 50:473–80. 10.1177/0004867415589793 26113745 PMC4843086

[166] Yu DX Di Giorgio FP Yao J Marchetto MC Brennand K Wright R. Modeling hippocampal neurogenesis using human pluripotent stem cells. Stem Cell Reports. (2014) 2:295–310. 10.1016/j.stemcr.2014.01.009 24672753 PMC3964286

[167] Hong S Yi JH Lee S Park CH Ryu JH Shin KS. Defective neurogenesis and schizophrenia-like behavior in PARP-1-deficient mice. Cell Death Dis. (2019) 10:943. 10.1038/s41419-019-2174-0 31819047 PMC6901579

[168] Osumi N Guo N. Impaired neurogenesis as a risk factor for schizophrenia and related mental diseases. In: Seki T, Sawamoto K, Parent JM, Alvarez-Buylla A, editors. Neurogenesis in the Adult Brain II. Tokyo: Springer (2011). p. 111–31. 10.1007/978-4-431-53945-2_6

[169] Avraham HK Jiang S Fu Y Rockenstein E Makriyannis A Zvonok A. The cannabinoid CB2 receptor agonist AM1241 enhances neurogenesis in GFAP/Gp120 transgenic mice displaying deficits in neurogenesis. Br J Pharmacol. (2014) 171:468–79. 10.1111/bph.12478 24148086 PMC3904265

[170] Molina-Holgado F Rubio-Araiz A García-Ovejero D Williams RJ Moore JD Arévalo-Martín Á. CB2 cannabinoid receptors promote mouse neural stem cell proliferation. Eur J Neurosci. (2007) 25:629–34. 10.1111/j.1460-9568.2007.05322.x 17328768

[171] Palazuelos J Ortega Z Díaz-Alonso J Guzmán M Galve-Roperh I. CB2 cannabinoid receptors promote neural progenitor cell proliferation via mTORC1 signaling. J Biol Chem. (2012) 287:1198–1209. 10.1074/jbc.M111.291294 22102284 PMC3256884

[172] Morais M Patrício P Mateus-Pinheiro A Alves ND MacHado-Santos AR Correia JS. The modulation of adult neuroplasticity is involved in the mood-improving actions of atypical antipsychotics in an animal model of depression. Transl Psychiatry. (2017) 7:e1146. 10.1038/tp.2017.120 28585931 PMC5537642

[173] Leucht S Cipriani A Spineli L Mavridis D Örey D Richter F Samara M. Comparative efficacy and tolerability of 15 antipsychotic drugs in schizophrenia: A multiple-treatments meta-analysis. Lancet. (2013) 382:951–62. 10.1016/S0140-6736(13)60733-3 23810019

